# Drug-associated hearing impairment in children: a disproportionality analysis of the FDA adverse event reporting system

**DOI:** 10.3389/fphar.2025.1532461

**Published:** 2025-06-26

**Authors:** Jinfeng Liu, Junyi Tan, Qinli Xiao, Yingtao Bai, En Chang, Chun Su, Yuxun Wei, Hu Zhong, Wei Wei

**Affiliations:** ^1^ Department of Pharmacy, People’s Hospital of Zhongjiang County, Deyang, Sichuan, China; ^2^ Department of Rehabilitation, People’s Hospital of Zhongjiang County, Deyang, Sichuan, China

**Keywords:** drug-associated hearing impairment, children, disproportionality, FAERS, pharmacovigilance

## Abstract

**Objective:**

Drug-associated hearing impairment has a serious impact on children’s quality of life and poses a significant public health burden. However, there is a lack of large-scale population-based studies of medication-associated hearing impairment in children. The aim of this study was to hypothesize about medications through data mining in order to assess the potential risk of these medications increasing hearing impairment in children.

**Methods:**

We extracted and analyzed reports on drugs linked to hearing impairment in children from the FDA Adverse Event Reporting System (FAERS). To assess the relationship between drugs and hearing impairment in children, we performed a disproportionality study utilizing the proportional reporting ratio (PRR) and reporting odds ratio (ROR). Concurrently, we conducted comparisons with medicine labels to identify medications that, although not now indicating hearing impairment in their labels, may possibly pose risks of hearing impairment in children.

**Results:**

In the FAERS database, there are 1,884 reports of AE related to hearing impairment in children. The top three medications with the highest ROR were vinblastin [N = 6 cases, ROR = 86.72 (34.15–220.19)], risedronate [N = 3 cases, ROR = 73.59 (20.24–267.63)], and amikacin [N = 11 cases, ROR = 71.31 (36.40–139.72)]. The top 3 drugs with the highest number of reports were carboplatin [N = 125 cases, ROR = 18.41 (15.27–22.21)], cisplatin [N = 78 cases, ROR = 31.24 (24.59–39.70)], and vincristine [N = 56 cases, ROR = 6.32 (4.83–8.27)]. Based on drug labeling, 48% drugs (27/56) were classified as potentially ototoxic.

**Conclusion:**

Our findings suggest that nearly half of the 56 drugs linked to hearing impairment signals in children are not currently labeled with ototoxicity warnings. Consequently, further research is required to evaluate the association of these medicines with this risk.

## Introduction

Ototoxicity is one of the most common adverse drug reactions, with symptoms including hearing loss and vestibular dysfunction, which have a serious impact on patients’ quality of life and have become a significant public health burden (Tanoshima et al., 2019). Vestibular dysfunction increases the risk of falls (Osoba et al., 2019), and both hearing and vestibular dysfunction are strongly associated with cognitive decline (Hanes and McCollum, 2006; Loughrey et al., 2018; Rizk et al., 2020b). Recognizing medication-induced ototoxicity is critical for healthcare providers in many areas (Rizk et al., 2020a). In an era of increasing polypharmacy, the negative public health impact of drug-induced hearing impairment cannot be ignored.

The impact and severity of ototoxicity may vary widely depending on pharmacology and individual patient risk factors (Rizk et al., 2020a). In addition, the consequences of hearing loss in children compared to adults are multifaceted and particularly affect patients treated at a young age. These consequences include impairments in speech and language acquisition, psychosocial and cognitive development, and educational and occupational achievement (Gurney et al., 2007; Schreiber et al., 2014; Olivier et al., 2019). Therefore, the effective prevention of hearing impairment due to ototoxic medications is becoming an area of great interest (Heinemann et al., 2020).

A few drugs are strongly associated with ototoxicity (e.g., aminoglycosides, labeled diuretics, platinum-based chemotherapeutic agents, etc.). However, more potentially ototoxic drugs may remain undiscovered (Rizk et al., 2020a). The FDA Adverse Event Reporting System (FAERS) is a database used to collect information about spontaneously reported adverse drug events. Because of its large volume of data, diversity of data information, and free public access, it is often used by pharmacovigilance experts in studies of adverse drug event signal mining to detect potential safety signals (Sakaeda et al., 2013).

This study aimed to identify drugs suspected of causing hearing impairment in children, utilizing the FAERS database. This study may provide data on medications that need to be studied further to determine the potential risk of these medications for hearing impairment in children.

## Methods

### Data source

This was a retrospective study utilizing the FAERS database, a database of spontaneously reported adverse events (AEs) designed to support post-marketing safety surveillance of drugs and biologics (Shen et al., 2019). We downloaded and extracted all data from the FDA website (fis.fda.gov/extensions/FPD-QDE-FAERS/FPD-QDE-FAERS.html) from the first quarter of 2014 to the second quarter of 2024. We then transferred the data to MySQL 8.0 for further analysis. In addition, FAERS data were updated quarterly, so there were duplicate reports. According to FDA recommendations, data de-duplication is performed prior to data analysis (Sakaeda et al., 2013). This procedure entailed choosing the most current FDA_DT when CASEID matched, and selecting the higher PRIMARYID when both CASEID and FDA_DT matched. Furthermore, our analysis was limited to AE reports from individuals aged 0–17 years.

### Adverse events and drug identification

The FAERS database adheres to the International Safety Reporting Guidelines outlined by the International Conference on Harmonization (ICH E2B). The classification and uniformity of ADEs in the FAERS data are based on the Medical Dictionary of Regulatory Activities (MedDRA) (Brown et al., 1999). The MedDRA nomenclature is organized into five levels: the System Organ Classifications (SOC), the High-Level Group Term (HLGT), the High-Level Term (HLT), the Preferred Term (PT), and the Lowest Level Term (LLT) (Mascolo et al., 2021). In addition, this can be achieved by adopting the Standardized MedDRA Query (SMQ) approach (Brown et al., 1999), where algorithms are used to combine several PTs to identify unique clinical syndromes. The definition of hearing impairment (code:20000171) in this study utilizes the definition of SMQ. Supplementary Table S1 displays the PTs associated with hearing impairment. To improve the accuracy of the analysis, our study only included AE reports for which the drug was the primary suspect.

### Drug classification

We reviewed the USA (https://www.accessdata.fda.gov/scripts/cder/daf/) and China (https://www.yaozh.com/) databases to identify medications previously recognized as ototoxic. Drug labeling serves to furnish healthcare providers and patients with thorough and precise information regarding medication use, thereby ensuring safety and efficacy (Wu et al., 2022). In this study, a drug was classified as “known” ototoxic if the side effect was indicated on the drug label. Otherwise, it was classified as a “potential” ototoxic agent.

### Data mining

This study used disproportionality analysis, also known as case/non-case analysis, which is one of the most common methods of detecting AE signals in pharmacovigilance. pharmacovigilance (Almenoff et al., 2007). The general principle is that we consider an AE signal to be generated when the reported rate of a specific AE for a particular drug is significantly higher than the background frequency in the database and meets certain criteria or thresholds. In this study, frequentist methods [reporting odds ratio (ROR) (van Puijenbroek et al., 2002) and proportional reporting ratio (PRR) (Evans, Waller, and Davis, 2001)] were used to identify potential AE signals associated with drug-hearing impairment. To improve the accuracy of the analysis, a meaningful AE signal was considered to be generated when both of these algorithms produced an AE signal. The formulas and thresholds for the two algorithms are shown in Table 1. All data processing was performed using MYSQL 8.0, Navicat Premium 16, and Microsoft Excel 2021.

**TABLE 1:** Two major algorithms used for signal detection.

Algorithms	Equation	Criteria
ROR	ROR = ad/b/c	lower limit of 95% CI>1, N≥3
	95%CI = e^ln(ROR)±1.96(1/a+1/b+1/c+1/d)^ ^0.5^	
PRR	PRR = a (c + d)/c/(a+b)	PRR≥2, χ^2^ ≥ 4, N≥3
	χ^2^ = [(ad-bc)^2^](a+b + c + d)/[(a+b)(c + d)(a+c)(b + d)]	

Equation: a, number of reports containing both the target drug and target adverse drug reaction; b, number of reports containing other adverse drug reaction of the target drug; c, number of reports containing the target adverse drug reaction of other drugs; d, number of reports containing other drugs and other adverse drug reactions. 95%CI, 95% confidence interval; N, the number of reports; χ^2^, chi-squared.

## Results

### Descriptive analysis

Between the first quarter of 2014 and the second quarter of 2024, the FAERS database documented 1,884 instances of childhood hearing impairment. A few cases were missing a minor amount of baseline information, leading to their classification as unknown. Table 2 presents the specific characteristics of children with drug-related hearing impairment. There were equal numbers of males (42.73%) and females (42.89%) among children with drug-related hearing impairment. The mean age was 8.34 ± 5.99 years. Hospitalization accounted for 21.71% of the reported drug-induced hearing impairment in children and deaths accounted for 2.07%. In addition, the country from which the most reports originated was the United States (35.83%), followed by Canada (14.28%) and France (8.39%). The frequency of reports of children with drug-induced hearing impairment has generally been rising over time.

**TABLE 2:** Clinical characteristics of children with drug-associated hearing impairment from the FAERS database (January 2014 to June 2024).

Characteristics	Subgroups	Case number, n	Case proportion, %
Number of events		1,884	
Gender	Female	808	42.89
	Male	805	12.24
	Unknown	271	75.54
Age	0–1 year	382	20.28
	2–5 years	347	18.42
	6–12 years	514	27.28
	13–17 years	641	34.02
Reporter	Consumer	538	28.56
	Physician	475	25.21
	Health professional	394	20.91
	Other health-professional	329	17.46
	Pharmacist	74	3.93
	Lawyer	30	1.59
	Unknown	4	0.21
Reported Countries (Top 5)	America	675	35.83
	Canada	269	14.28
	France	158	8.39
	Germany	126	6.69
	Great Britain	116	6.16
Year	2023-2024q2	231	12.26
	2020–2022	684	36.31
	2017–2019	517	27.44
	2014–2016	452	23.99
Serious Outcome	Other Serious (Important Medical Event)	1,361	72.24
	Hospitalization - Initial or Prolonged	409	21.71
	Disability	280	14.86
	Congenital Anomaly	177	9.39
	Life-Threatening	64	3.40
	Death	39	2.07
	Required Intervention to Prevent Permanent Impairment/Damage	7	0.37
	Unknown	188	9.98

Abbreviations: FAERS, United States Food and Drug Administration Adverse Event Reporting System; q2, quarter 2.

### Disproportionality analysis

**FIGURE 1:**
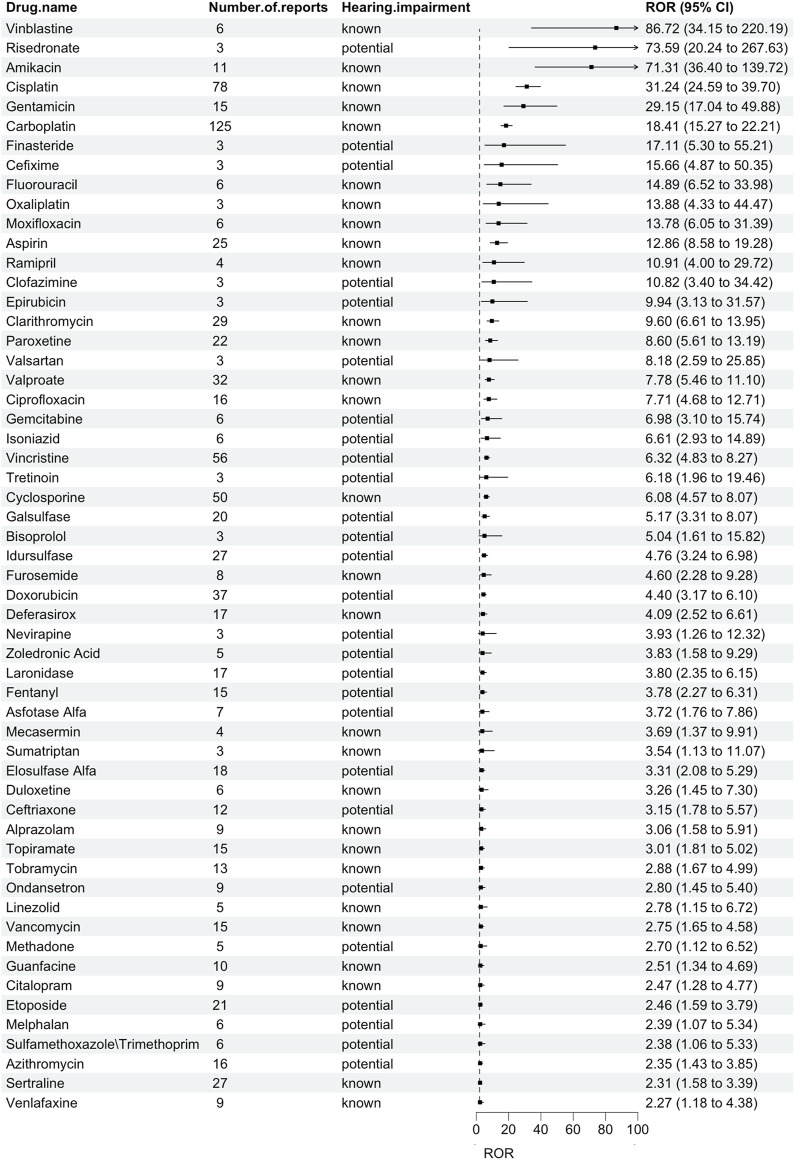
Drugs related to hearing impairment in children based on ROR.

We discovered 56 pharmaceuticals in the FAERS database linked to hearing impairment in children (Figure 1). Vinblastin [N = 6, ROR = 86.72 (34.15–220.19)], risedronate [N = 3, ROR = 73.59 (20.24–267.63)], and amikacin [N = 11, ROR = 71.31 (36.40–139.72)] were the top three drugs with the highest ROR. The top 3 drugs with the highest number of reports were carboplatin [N = 125, ROR = 18.41 (15.27–22.21)], cisplatin [N = 78, ROR = 31.24 (24.59–39.70)], and vincristine [N = 56, ROR = 6.32 (4.83–8.27)]. According to drug labeling, 29 drugs were categorized as known ototoxic agents, while 27 drugs were categorized as potentially ototoxic agents.

Table 3 lists the number of AEs recorded for each medication class based on the Anatomical Therapeutic Chemical (ATC) classification. Anti-infectives for systemic use, antineoplastic and immunomodulating agents, and nervous system were the three drug classes with the highest reporting rates. Reports related to hearing impairment in children were commonly reported in FAERS at 26.79%, 21.43%, and 19.64%, respectively. Among these, paroxetine [N = 22, ROR = 8.60 (5.61–13.19)] had the highest ROR among the medications for the nervous system, vinblastine had the highest ROR among the antineoplastic and immunomodulating agents, and amikacin had the highest ROR among the anti-infectives for systemic use in children with hearing impairment.

**TABLE 3:** Drug-associated with hearing impairment in children based on ATC classification.

ATC	Number of drugs (N = 56)	%
Alimentary tract and metabolism (A)	6	10.71
Blood and blood forming organs (B)	1	1.79
Cardiovascular system (C)	5	8.93
Dermatologicals (D)	2	3.57
Systemic hormonal preparations, excl. Sex hormones and insulins (H)	1	1.79
Anti-infectives for systemic use (J)	15	26.79
Antineoplastic and immunomodulating agents (L)	12	21.43
Musculo-skeletal system (M)	2	3.57
Nervous system (N)	11	19.64
Various (V)	1	1.79

Abbreviations: ATC, Anatomical Therapeutic Chemical.

## Discussion

This study utilized 10 years of data from the FAERS database to identify suspected medications that may be associated with hearing impairment in children. We identified 1,884 reports of associated AE. Furthermore, we found 56 medications linked to hearing impairment in children in the FAERS database. Among them, the categories with the largest number of reports were anti-infectives for systemic use, antineoplastic and immunomodulating agents, and nervous system. The three medications with the greatest ROR were vinblastine, risedronate, and amikacin. It is noteworthy that approximately 48% of the drugs showed a potential hearing impairment in children and were not clearly identified on the drug labeling as having associated adverse effects.

In an age of rising polypharmacy, severe medication responses such ototoxicity pose considerable public health concerns. This study using disproportionality analysis to identify safety signals linking medications and hearing impairment in children. In our study, we first identified some drugs that are clearly ototoxic, such as platinum-based chemotherapeutic agents, aminoglycoside antibiotics, and vancomycin (Freyer et al., 2020; Diepstraten et al., 2021; Lestner et al., 2016). The ototoxicity induced by these drugs is mainly characterized by cochlear and vestibular toxicity, and the contribution of aminoglycoside antibiotics to the risk of ototoxicity in children is particularly significant. According to the World Health Organization, among the preventable causes of hearing loss in children, about 4% of ototoxicity cases are attributed to the use of ototoxic drugs such as aminoglycosides in pregnant women and infants (Lieu et al., 2020). Clinical experience and previous studies have shown that clinical experience and previous studies suggest that aminoglycoside antibiotics may induce cochlear toxicity and/or vestibular toxicity in some pediatric patients (Lanvers-Kaminsky et al., 2017). Cochlear toxicity is caused by hair cell rupture and can lead to neurologic hearing impairment and even blindness in severe cases (Jiang, Karasawa, and Steyger, 2017). This is in agreement with the results of the present study, where AE signals for hearing impairment were detected for amikacin, gentamicin, and tobramycin, and amikacin and gentamicin were the two drugs with the highest ROR for hearing impairment among the antimicrobials.

Platinum-based drugs are commonly used as chemotherapeutic agents for the treatment of childhood cancers, and 60% of children treated with cisplatin develop permanent bilateral hearing impairment (Knight, Kraemer, and Neuwelt, 2005; Knight et al., 2007). In the present study, we found that carboplatin and cisplatin were the two drugs with the highest number of reports, and oxaliplatin also showed AE signals of potential hearing impairment. This result is in line with other research and indicates that there may be some variation in hearing impairment brought on by platinum compounds, with oxaliplatin being the least ototoxic and cisplatin being the most ototoxic (Grewal et al., 2010; Romano et al., 2020).

Aspirin is one of the most commonly used antipyretic and analgesic drugs for children. Because children with Kawasaki disease need to take aspirin for a long period of time and in large quantities, it is easy to have headaches, dizziness, tinnitus, visual hearing loss, and other symptoms, known as the salicylic acid reaction, whose mechanism may be the vasoconstriction of vasculature supplying the helix caused by salicylic acid, and the children may have monaural or binaural hearing loss, but if the drug is stopped in a timely manner, most of them can be restored (Salvi et al., 2021). Aspirin has been demonstrated to substantially diminish the risk of hearing impairment produced by gentamicin (Chen et al., 2007). Nonetheless, owing to the distinct processes of ototoxicity associated with cisplatin and gentamicin, research indicates that aspirin does not safeguard hearing in individuals undergoing cisplatin treatment (Crabb et al., 2017). Despite the variability in clinical study outcomes, we must be cautious regarding the potential risk of hearing impairment associated with aspirin in children.

Antidepressants are widely known for causing ototoxicity due to tinnitus and positional vertigo (Clewes, 2012), and the present study also found multiple antidepressants associated with ototoxicity in children. In addition, iron chelators have been strongly associated with neurologic hearing loss at high frequencies (Osma et al., 2015), and our study also observed a significant proportion of AE reports involving deferasirox-induced hearing impairment. The current study indicates that valsartan and bisoprolol may possess ototoxic potential, corroborating findings from a prior review that identified angiotensin-converting enzyme inhibitors and angiotensin II receptor blockers as potentially ototoxic agents (Cianfrone et al., 2011). In a related study, decreased beta receptor function may lead to hearing deficits (Al-Ghamdi et al., 2018). Hearing impairments have been linked to the use of medications that enhance peripheral vascularization. Additionally, hypertension patients may experience tinnitus as a result of taking medications that stimulate the renin-angiotensin system or raise peripheral vascular tone (β-blockers) (Borghi et al., 2005).

Notably, the data-mining results of this study revealed that approximately 60% of drugs with potential pediatric hearing impairment signals do not mention this adverse effect in their drug labeling. These drugs include neurologic drugs such as valproate and sertraline, and cardiovascular drugs such as valsartan and bisoprolol. The association of these drugs with ototoxicity has not been well documented, and there are only a few case reports suggesting that these drugs may cause hearing impairment (Armon et al., 1990; Hori et al., 2003; Yeap et al., 2014). In addition, this study found that drugs used to treat mucopolysaccharide storage disease (e.g., galsulfase, idursulfase, laronidase, and elosulfase alfa), and the third-generation bisphosphonates risedronate and zoledronic acid, used to treat hearing loss due to pediatric sclerosis, have also been associated with ototoxicity signaling correlations (Quesnel et al., 2012). Although these signals may be more associated with primary disease (Ago et al., 2024; Karosi, Szekanecz, and Sziklai, 2009).

Certain limitations of this study should be acknowledged while analyzing the results. Firstly, because the total number of patients treated with a particular medication was not available, we were unable to calculate the incidence of drug-induced hearing impairment for each medication. Secondly, FAERS is constrained by the initiative, precision, and promptness with which physicians, other healthcare professionals, and consumers report adverse events. This may lead to potential misreporting and underreporting. Finally, the study design only allowed for the monitoring of safety signals, and therefore future studies are needed to validate associations and confirm causality between them (Fusaroli et al., 2024). Despite these limitations, our study will provide useful guidance to healthcare providers and the public, reveal potential medications associated with hearing impairment in children, and provide valuable data to support the monitoring and clinical application of relevant medications in the future.

## Conclusion

This pharmacovigilance study explored reports of AEs associated with pediatric hearing impairment in the FAERS database. Our research indicated that almost 48% of the 56 examined pharmaceuticals with possible safety signals for pediatric hearing impairment did not mention ototoxicity as an adverse effect in their labeling. Consequently, further research is required to evaluate the association of these drugs with this risk.

## Data Availability

The raw data supporting the conclusions of this article will be made available by the authors, without undue reservation.
