# Supplementary material for: Drug-associated hearing impairment in children: a disproportionality analysis of the FDA adverse event reporting system
Source: Front Pharmacol. 2025 Jun 26;16:1532461. doi: 10.3389/fphar.2025.1532461 (PMC12241047; doi:10.3389/fphar.2025.1532461)
Supplement: Supplementary file 1 [file Table1.docx]

Supplementary Table 1. SMQ and PT associated with hearing impairment

| SMQ | PT | |
| --- | --- | --- |
| Hearing impairment (SMQ) [20000171] | Acoustic stimulation tests abnormal [10000526] |  |
|  | Acquired deaf mutism [10087739] |  |
|  | Altered pitch perception [10075083] |  |
|  | Audiogram abnormal [10003761] |  |
|  | Auditory disorder [10003778] |  |
|  | Auditory recruitment [10003789] |  |
|  | Autophony [10048827] |  |
|  | Barotitis media [10004129] |  |
|  | Bone anchored hearing aid implantation [10070723] |  |
|  | Cochlea implant [10009830] |  |
|  | Conductive deafness [10010280] |  |
|  | Deafness [10011878] |  |
|  | Deafness bilateral [10052556] |  |
|  | Deafness neurosensory [10011891] |  |
|  | Deafness occupational [10011893] |  |
|  | Deafness permanent [10011894] |  |
|  | Deafness transitory [10011900] |  |
|  | Deafness unilateral [10048812] |  |
|  | Diplacusis [10013032] |  |
|  | Dysacusis [10049712] |  |
|  | Electrocochleogram abnormal [10014399] |  |
|  | Eustachian tube disorder [10061462] |  |
|  | Eustachian tube dysfunction [10015543] |  |
|  | Eustachian tube obstruction [10015544] |  |
|  | Haematotympanum [10063013] |  |
|  | Hearing aid therapy [10075385] |  |
|  | Hearing therapy [10087034] |  |
|  | Hyperacusis [10020559] |  |
|  | Hypoacusis [10048865] |  |
|  | Middle ear adhesions [10027582] |  |
|  | Middle ear effusion [10062545] |  |
|  | Middle ear inflammation [10065838] |  |
|  | Misophonia [10079388] |  |
|  | Mixed deafness [10027757] |  |
|  | Myringosclerosis [10087692] |  |
|  | Neonatal deafness [10080897] |  |
|  | Neonatal hypoacusis [10080902] |  |
|  | Neurosensory hypoacusis [10067587] |  |
|  | Noninfective myringitis [10078830] |  |
|  | Ossicle disorder [10061327] |  |
|  | Otoacoustic emissions test abnormal [10063643] |  |
|  | Otosclerosis [10033103] |  |
|  | Ototoxicity [10033109] |  |
|  | Paracusis [10085733] |  |
|  | Presbyacusis [10036626] |  |
|  | Rinne tuning fork test abnormal [10039191] |  |
|  | Sudden hearing loss [10061373] |  |
|  | Tinnitus [10043882] |  |
|  | Tinnitus retraining therapy [10084652] |  |
|  | Tympanic membrane atrophic [10045208] |  |
|  | Tympanic membrane disorder [10062218] |  |
|  | Tympanic membrane perforation [10045210] |  |
|  | Tympanometry abnormal [10045215] |  |
|  | Tympanosclerosis [10045218] |  |
|  | Weber tuning fork test abnormal [10047878] |  |

Abbreviations: SMQ, Standardized Medical Dictionary for Regulatory Activiti Query; PT, preferred term.
